# The Dilemma of Misclassification Rates in Senior Patients With Prostate Cancer, Who Were Treated With Robot-Assisted Radical Prostatectomy: Implications for Patient Counseling and Diagnostics

**DOI:** 10.3389/fsurg.2022.838477

**Published:** 2022-02-16

**Authors:** Nikolaos Liakos, Joern H. Witt, Pawel Rachubinski, Sami-Ramzi Leyh-Bannurah

**Affiliations:** Prostate Center Northwest, Department of Urology, Pediatric Urology and Uro-Oncology, St. Antonius-Hospital, Gronau, Germany

**Keywords:** frailty, elderly patients, age factors, prostate cancer, upgrading, RARP, propensity score matching

## Abstract

**Objectives:**

There is a recent paradigm shift to extend robot-assisted radical prostatectomy (RARP) to very senior prostate cancer (PCa) patients based on biological fitness, comorbidities, and clinical PCa assessment that approximates the true risk of progression. Thus, we aimed to assess misclassification rates between clinical vs. pathological PCa burden.

**Materials and Methods:**

We compared senior patients with PCa ≥75 y (*n* = 847), who were propensity score matched with younger patients <75 y (*n* = 3,388) in a 1:4 ratio. Matching was based on the number of biopsy cores, prostate volume, and preoperative Cancer of the Prostate Risk Assessment (CAPRA) risk groups score. Multivariable logistic regression models (LRMs) predicted surgical CAPRA (CAPRA-S) upgrade, which was defined as a higher risk of the CAPRA-S in the presence of lower-risk preoperative CAPRA score. LRM incorporated the same variables as propensity score matching. Moreover, patients were categorized as low-, intermediate-, and high-risk, preoperative and according to their CAPRA and CAPRA-S scores.

**Results:**

Surgical CAPRA risk strata significantly differed between the groups. Greater proportions of unfavorable intermediate risk (39 vs. 32%) or high risk (30 vs. 28%; *p* < 0.001) were observed. These proportions are driven by greater proportions of International Society of Urological Pathology (ISUP) Gleason Grade Group 4 or 5 (33 vs. 26%; *p* = 0.001) and pathological tumor stage (≥T3a 54 vs. 45%; *p* < 0.001). Increasing age was identified as an independent predictor of CAPRA-S-based upgrade (age odds ratio [OR] 1.028 95% CI 1.02–1.037; *p* < 0.001).

**Conclusion:**

Approximately every second senior patient has a misclassification in (i.e., any up or downgrade) and each 4.5th senior patient specifically has an upgrade in his final pathology that directly translates to an unfavorable PCa prognosis. It is imperative to take such substantial misclassification rates into account for this sensitive PCa demographic of senior men. Future prospective studies are warranted to further optimize PCa workflow and diagnostics, such as to incorporate modern imaging, molecular profiling and implement these into biopsy strategies to identify true PCa burden.

## Introduction

Prostate cancer (PCa) is the second most common malignant tumor entity in men, especially in industrialized countries (1), and may present in a variety of oncological profiles, varying from insignificant to highly aggressive diseases.

As the diagnostic tools for PCa evolve, there will be an increase of patient numbers needing counseling with regard to possible treatment options in the upcoming years (1, 2). Among these patients, there will be many senior men over the age of 75 years.

Our series was one of the first to demonstrate that senior age is not a contraindication for local treatment, such as robot-assisted radical prostatectomy (RARP) (3, 4). Specifically, our results demonstrated that senior age is not inherently a risk factor for unfavorable prognosis and that sufficient functional outcomes can be achieved that are highly comparable to younger patients, such as urinary continence recovery. Thus, we contributed to a paradigm shift that selects senior patients who can be in fact counseled for local therapies, such as RARP (3, 4).

However, consistent with several studies developing prediction tools for upgrading and upstaging, when biopsy vs. final histopathology was compared, senior age appeared indeed to have more unfavorable pathology and our own observations indicated greater misclassification between clinical vs. pathological stages. This phenomenon of misclassification likely explains findings of previous studies that reported rather higher rates of biochemical recurrence or even metastatic progression in senior men (5–7), since the studies adjusted for clinical parameters. In fact, such contradicting findings in combination with a rather dated concept to derive life expectancy based on demographics instead at the individual level supported the widely adopted notion to refrain from offering surgical local treatment to senior patients with PCa since the beneficiary potential of RARP appeared compromised (3, 8–11). Alternatively, these patients will be offered radiotherapy or administration of an androgen deprivation therapy (12), both options potentially associated with high morbidity and comparably ineffective treatment of possible Pre-existing urinary tract symptoms (13). Moreover, guidelines, such as the United States Preventive Services Taskforce (USPSTF), recommend no PCa screening in men ≥70 years based on clinical findings, which might lead to further aggravation of the aforementioned misclassification problem in senior aged men. It is of note that senior age sample sizes, which were treated with RARP, are accordingly very small and that most series did not rely on adjustment according to pathological characteristics or that methodological implications apply, such as relying on univariable analyses.

We hypothesized that even after multivariable adjustment, there is a substantial misclassification, i.e., unfavorable PCa upgrade at RARP in senior patients compared to younger patients, which might delay treatment or even preclude senior patients from local treatment.

To examine this potentially detrimental misclassification phenomenon in senior men, we applied pre and postoperative Cancer of the Prostate Risk Assessment risk groups (CAPRA and CAPRA-S, respectively) (14), which represent multivariable scores that rely on clinical and pathological variables, respectively. Based on CAPRA vs. CAPRA-S risk scores, we analyzed the rates of an upgrade at RARP between senior patients with PCa aged ≥75 years vs. younger patients with PCa <75 years.

## Materials and Methods

Overall, 13,765 consecutive patients with PCa were identified, who had complete pathological data and were treated with RARP and extended pelvic lymph node dissection at the Prostate Cancer Center Northwest, Gronau, Germany, between May 2006 and December 2019. All patient data were gathered prospectively.

Patients with suspected metastases were excluded and required complete clinico-pathological data to derive both, the University of California, San Francisco (UCSF) preoperative (15) and surgical Cancer of the Prostate Risk Assessment scores (CAPRA and CAPRA-S, respectively) (14).

The preoperative CAPRA incorporates age, clinical tumor stage, Pre-surgical prostate-specific antigen (PSA), biopsy Gleason score, and percentage of positive biopsy cores (15). The final CAPRA score ranges from 0 to 10, with 0–2 representing low risk (LR), 3–5 intermediate risk (IR), and 6–10 high risk (HR). Similarly, the CAPRA-S incorporates pre-surgical PSA, pathological Gleason score, surgical margin status, extracapsular extension, seminal vesicle invasion, and lymph node invasion (16). Patients were stratified according to age groups <75 vs. ≥75 years. The final CAPRA-S score ranges from 0 to 12, with 0–2 representing LR, 3–5 IR, and 6–12 HR. For both, CAPRA and CAPRA-S, each two-point increase in score indicates an approximately 2-fold increase in the risk of PCa recurrence or secondary treatment.

### Propensity Score Matching

We used propensity score matching at a 4:1 ratio to create similar cohorts between patients with RARP <75 vs. ≥75 years. We ensured that matching improved the balance between the aforementioned age groups by checking the standardized mean differences. Propensity score matching variables consisted of the same variables that were used for the multivariable logistic regression analyses (LRM): age, year of surgery, the number of biopsy cores, and preoperative CAPRA score.

### Statistical Analyses

The chi-square test was used for categorical and *t*-test for continuous variables. Proportions of the respective CAPRA and CAPRA-S risk strata (14), i.e., LR, IR, and HR, were calculated and compared. The CAPRA-S-based upgrade was defined as a lower risk at preoperative CAPRA despite harboring a higher risk CAPRA-S at RARP, for example, preoperative CAPRA LR vs. CAPRA-S IR or even CAPRA-S HR.

Logistic regression model aimed to predict CAPRA-S-based upgrade at RARP and was adjusted for age, year of surgery, the number of biopsy cores sampled, and preoperative CAPRA score. It is important to note that a CAPRA-S-based upgrade cannot be observed in men categorized as preoperative CAPRA HR. Accordingly, LRM was restricted to patients with preoperative CAPRA LR or IR. LRM was additionally performed after propensity score matching.

All tests were two-sided with a statistical significance set at *p* < 0.05. Analyses were performed with the statistical package for R (R Foundation for Statistical Computing, version 3.2.2).

## Results

### Overview Comparison of Patient Characteristics

Clinico-pathological characteristics of 847 senior patients with PCa ≥75 years, who were propensity score matched with 3,388 younger patients with PCa <75 years (4:1 ratio) are presented in Table 1. The respective median age was 76 (interquartile range [IQR] 75–77) vs. 65 (IQR 60–69) years.

**Table 1:** Clinicopathological characteristics of 847 senior prostate cancer patients ≥ 75 yrs., who are propensity score matched with 3,388 (4:1 ratio) younger prostate cancer patients <75 yrs., according to preoperative CAPRA score, number of biopsy cores and prostate weight.

		**After propensity score matching^a^**			
**Value**	**Patients** **<** **75 yrs. (*****n*** **=** **3,388)**		**Patients** **≥75 yrs. (*****n*** **=** **847)**		***p*-value**
Age, years, median (IQR)	65	(60–69)	76	(75–77)	<0.001
Preoperative serum PSA, ng/ml, median (IQR)	8.5	(5.9–14)	7.8	(5.3–11)	<0.001
Number of biopsy cores, median (IQR)	12	(10–13)	12	(10–14)	0.2
Percent of positive biopsy cores, median (IQR)	37%	(18–58%)	33%	(20–51%)	0.59
Prostate weight, *g*, median (IQR)	46	(37–61)	47	(36–63)	0.35
Clinical tumor stage, *n* (%)					0.08
cT1	1,881	55%	462	54%	
cT2	1,372	40%	352	42%	
cT3a or more	135	4.0%	33	4%	
Gleason score pattern (primary or secondary), *n* (%)					0.001
No gleason 4 or 5 pattern present	1,000	29%	194	23%	
Gleason 4 or 5 pattern secondary	1,075	32%	287	34%	
Gleason 4 or 5 pattern primary	1,313	39%	366	43%	
Pathological ISUP grade, *n* (%)					<0.001
1	751	22%	118	14%	
2	1,042	31%	247	29%	
3	716	21%	201	24%	
≥4	879	26%	281	33%	
Pathological tumor stage, *n* (%)					<0.001
pT2	1,860	55%	391	46%	
pT3a or more	1,528	45%	456	54%	
Lymph node invasion, *n* (%)	489	14%	128	15%	0.65
Positive surgical margins, *n* (%)	381	11%	98	12%	0.84
CAPRA risk group, *n* (%)					0.001
Low risk	717	21%	151	18%	
Intermediate risk	1,661	49%	478	56%	
High risk	1,010	30%	218	26%	
CAPRA-S risk group, *n* (%)					<0.001
Low risk	1,340	39%	266	31%	
Intermediate risk	1,099	32%	329	39%	
High risk	949	28%	252	30%	
Comparison of CAPRA and CAPRA-S scores, *n* (%)					<0.001
Downgrading at prostatectomy	1,053	31%	223	26%	
Concordant	1,934	57%	485	57%	
Upgrading at prostatectomy	401	12%	139	16%	

a *Matched by preoperative CAPRA score, number of biopsy cores and prostate weight*.

*CAPRA, Cancer of the Prostate Risk Assessment; CAPRA-S, the postsurgical Cancer of the Prostate Risk Assessment score; ISUP, International Society of Urological Pathology; IQR, interquartile range*.

Propensity score matching yielded virtually identical proportions of preoperative CAPRA risk strata between age groups, with LR, IR, and HR being 18–21%, 56–49%, and 26–30%, respectively. However, it is of note that minor differences were still observed in underlying clinical characteristics between ≥75 vs. <75 years, such as median PSA (7.8 vs. 8.5; *p* < 0.001), and primary biopsy Gleason pattern 4 or 5 (43 vs. 39%; *p* = 0.001).

Despite such virtually identical proportions of preoperative CAPRA risk strata, the RARP-derived CAPRA-S risk strata significantly differed in ≥75 vs. <75 years. Specifically, greater proportions of unfavorable IR (39 vs. 32%) or HR (30 vs. 28%; *p* < 0.001) were observed in senior patients with PCa. These proportions are driven by greater proportions of ISUP The Gleason Grade Group 4 or 5 (43 vs. 39%; *p* = 0.001) and pathological tumor stage (≥T3a 54 vs. 45%; *p* < 0.001) and aforementioned PSA, whereas modestly higher proportions of positive surgical margin (12 vs. 11%; *p* = 0.84) or nodal metastases (15 vs. 14%; *p* = 0.65) were observed but not statistically significant.

Comparison of preoperative CAPRA and RARP CAPRA-S to determine rates of CAPRA-S-based upgrade translated to higher rates of CAPRA-S upgrade in senior patients with PCa (16. vs. 12%; *p* < 0.001). Conversely, lower downgrade rates were observed in the same senior patients (26 vs. 31%; *p* < 0.001). Specifically, if analyses were restricted to propensity score-matched senior patients with a preoperative CAPRA LR or IR profile, rates of CAPRA-S upgrade in senior patients with PCa were even more distinct, with 22 vs. 17% (*p* < 0.001).

Finally, LRM, which were restricted to men with preoperative CAPRA LR or IR and were adjusted for age, number of biopsy cores sampled, prostate weight, and preoperative CAPRA score, revealed increasing age as an independent predictor of CAPRA-S-based upgrade at RARP (age OR 1.028 95% CI 1.02–1.037; *p* < 0.001, Table 2).

**Table 2:** Multivariable logistic regression model predicting CAPRA-S based prostate cancer risk upgrade at robot-assisted radical prostatectomy before and after propensity score matching.

**Before propensity score matching**					**After propensity score matching^a^**				
	**OR**	**95% CI**		***p*-value**		**OR**	**95% CI**		***p*-value**
**Age**	1.028	1.019	1.036	<0.001	**Age**	1.039	1.025	1.052	<0.001

a *Adjusted for age, total number of biopsy cores sampled, CAPRA-Score and prostate weight*.

To account for the introduction of multiparametric MRI of the prostate and MRI-ultrasound fusion biopsies since around 2015, we additionally performed analyses, which were restricted to recent time intervals 2015–2017 and ≥2018. Even in these most contemporary cohorts, senior age was associated with a higher risk of CAPRA-S-based upgrade at RARP in LRM (data not shown).

## Discussion

The demographic transition in industrialized countries will have a major effect on the burden of the urological departments in the next decades. An increasing number of senior patients will present themselves with newly diagnosed PCa and seek curative treatment (6). However, based on a rather stringent concept of 10 years life expectancy, which is mostly based on national demographic estimates and in compliance with present guidelines, many patients are precluded from local treatment, when such a patient is being consulted for treatment. This is despite recent series, which demonstrated that senior patients, who are treated with RARP, might experience low perioperative morbidity, achieve excellent results of cancer control, quality of life (QoL), and functional results (4, 13, 17, 18). Conversely, it is of utmost importance to consider watchful waiting strategies or active surveillance after carefully weighing risk and benefit and QoL (19).

Thus, the consultation needs to account for several aspects, the individual, his biological fitness, and his clinical PCa characteristics. As a prerequisite, potential misclassification rates must also be considered, particularly, if the treatment is potentially delayed and the window of opportunity of cure might be missed.

Our study yielded several important results:

First, the vast majority of our senior patients with PCa, who were treated with RARP, (82%, Table 1), had preoperative CAPRA IR or HR. This indicates adequate selection criteria for local treatment. However, a comparison of preoperative CAPRA LR with CAPRA-S LR within senior patients revealed substantial differences, i.e., 18 vs. 31%. This indicates misclassification, i.e., downgrading at RARP. This means that these patients might also have benefitted from other solutions than local treatment, such as active surveillance. However, it is of note that CAPRA LR is not as strict as widely adapted active surveillance criteria, such as European Association of Urology (EAU) or Prostate Cancer Research International Active Survaillance study (PRIAS) (20).

Similarly, a comparison of CAPRA HR with CAPRA-S HR within senior patients also reveals Non-negligible differences, 26–30% (Table 1). This indicates misclassification, i.e., upgrading at RARP. Taken together, these combined misclassification rates of 43% at senior age are rather astonishing since CAPRA and CAPRA-S each represent multivariable scores relying on clinical and pathological PCa characteristics, respectively, to denote oncological prognosis. However, the previous series also suggested that CAPRA and CAPRA-S might be further optimized by incorporating better metrics of tumor burden, such as Gleason quantification to optimize the prediction of recurrent disease or metastatic progression (21).

Moreover, the previous series suggested that senior age was an independent predictor for worse oncological and functional outcomes after radical prostatectomy (6). This point could not be confirmed in a previous study of our center (22), supporting the argument that local surgical treatment of PCa, potentially in the context of a multimodal setting, might confer a survival benefit. Therefore, senior patients should not be excluded from RARP, particularly, when accounting for current demographic trends toward an increasing number of senior PCa patients with unfavorable tumor characteristics (23).

Second, after matching, a comparison of senior patients with PCa ≥75 vs. <75 years, who were either CAPRA LR or IR, revealed higher rates of CAPRA-S-based upgrade at RARP (22 vs. 17%; *p* = 0.002, data not shown). It is very important to note that baseline characteristics in Table 1 represent comprehensively propensity score-matched data between both age groups, according to the number of biopsy cores sampled, prostate volume, and preoperative CAPRA score. Our findings demonstrate that age is an independent risk factor for CAPRA-S-based upgrade at RARP.

Moreover, it is also of note that a higher CAPRA-S score in senior patients was driven by ISUP Gleason Grade group 4 or 5 and/or extracapsular extension of the PCa. Both, the Gleason Grade group and tumor stage can be assessed with the multiparametric MRI (mpMRI) of the prostate. Unfortunately, many patients with LR PCa at biopsy are unlikely to receive further imaging prior to surgery (mpMRI), as it is not mentioned in the present guidelines and there is no reimbursement covered by health insurance in Germany. This may one of many factors contributing to a systematic underestimation of true PCa aggressiveness in senior men. This argument supports additionally the implementation of prostate MRI as a definite staging tool prior to consultation. Specifically, studies of Nordström et al. and Moore et al. demonstrated that the inclusion of MRI in longitudinal PCa biopsy and diagnostic pathways lead to a clear decrease of unnecessary re-biopsies and a higher yield of the clinical significant PCa (24, 25). Those pathways are likely to further improve if combined with deep learning-based analyses of imaging features, such as apparent diffusion coefficient (ADC) maps (26).

Third, our multivariable analyses confirmed age as an independent risk factor for CAPRA-S-based upgrade at RARP. Before and after propensity score matching, results were virtually identical: increasing age was associated with higher odds of CAPRA-S-based upgrade at RARP (matched OR 1.039, 95% 1.025–1.052; *p* < 0.001, Table 2). It is of note that several systematic biopsy cores were not statistically significant, further underlining the notion that certain senior patients are insufficiently sampled with current systematic biopsy strategies. Taken together, to mitigate greater susceptibility of misclassification rates in senior men and to account for tumor stage and potential extracapsular extension, mpMRI should be strongly considered at primary diagnosis if the patient has advanced age.

As we are in the middle of the COVID-19 pandemic, the trend of unfavorable characteristics of PCa will continue to develop, leading to a late diagnosis and a delayed treatment with histopathological signs of an advanced stage of cancer (27). We already observed a rise in the proportion of RARP patients with aggressive tumor characteristics in our daily practice. It is mandatory to at least evaluate to extend the indication of RARP to physically and cognitively fit senior patients that might be suitable for surgery (5, 28, 29).

It is important to note that a higher CAPRA-S risk profile, which denotes unfavorable pathology, is associated with a higher chance of adjuvant or salvage treatment. However, recent population-based data revealed that Post-prostatectomy treatment should be carefully evaluated in very senior men due to greater competing other-cause mortality rates compared to men, who were just treated with prostatectomy (30).

Due to our observed misclassification rates, particularly in senior men, it is necessary to not only rely on conventional clinico-pathological parameters but also account for any available preoperative staging information and host characteristics, such as age, cardiovascular, and cognitive fitness. Latter are rarely integrated into widely utilized prediction tools.

Our study has limitations: first of all, all senior patients who received RARP were selected and described as “fit for surgery” after thorough anesthesiological examination with a focus on cardiovascular and mental fitness. However, no dedicated geriatric assessment tools were regularly utilized. Second, all patients were treated in a high-volume center by very experienced surgeons. Therefore, the generalizability of our study findings is limited. Third, our data were derived from a single center.

Despite the matching, there are still important caveats. CAPRA and CAPRA-S do not account for highly variable differences of tumor burden and Gleason quantification that might occur within the same risk-strata. For the same reason, it is of note that CAPRA relies on the same clinical variables (PSA, biopsy Gleason grade, and clinical tumor stage) as the widely utilized D'Amico classification, and additionally includes age at diagnosis and percent of biopsy cores involved with cancer as a metric of cancer burden. These variables represent essential variables for multivariable predictions of misclassification (31). Thus, we relied on CAPRA to allow for a more precise risk stratification than the D'Amico classification system.

Moreover, as some series strongly indicate, there are potentially very distinct molecular patterns underlying PCa that result in an upgrading of the tumor, such as *de novo* development of high-grade tumors after biopsy, spatial heterogeneity from the same tumor focus, or clonal progression from low- to high-grade cancer (32–35). Moreover, a certain lead time is highly conceivable in senior PCa patients with regard to the untreated natural history of PCa. Finally, ultrasound quality might deteriorate due to a greater probability of hyperechogenic artifacts, such as calcifications, subsequently decreasing systematic biopsy performance.

## Conclusions

After a thorough multivariable adjustment, we observed that each 4.5th senior patient has an upgrade in his pathology and virtually every second senior patient has a misclassification in (i.e., any up or downgrade) that directly translates to PCa prognosis. Bearing in mind that select senior patients might greatly benefit from an adequate therapy, such as RARP, it is imperative to take such substantial misclassification rates into account for this PCa demographic, which will be continuously increasing and more relevant in the years to come. Future prospective studies are warranted to further optimize PCa workflow and diagnostics, utilizing tools, such as modern imaging, molecular profiling, and implementing them into new biopsy strategies to identify true PCa burden.

## Data Availability Statement

The original contributions presented in the study are included in the article/supplementary materials, further inquiries can be directed to the corresponding author.

## Ethics Statement

The studies involving human participants were reviewed and approved by the Institutional Review Board at the St. Antonius-Hospital, Gronau, approved the retrospective study design and access to the patients' medical records. All methods were carried out in accordance with the Declaration of Helsinki. Written informed consent was obtained from individual participants in the study. The patients/participants provided their written informed consent to participate in this study.

## Author Contributions

S-RL-B and NL had full access to all the data in the study, takes responsibility for the integrity of the data, the accuracy of the data analysis, participated in the study concept, and design. NL, S-RL-B, PR, and JW performed the acquisition of data. S-RL-B performed analysis, interpretation of data, and statistical analysis. NL participated in the drafting of the manuscript. S-RL-B and JW participated in the critical revision of the manuscript for important intellectual content, and done supervision. Administrative, technical, or material support was given by PR. All authors contributed to the article and approved the submitted version.

## Conflict of Interest

JW is a paid proctor and consultant for Intuitive Surgical and Board member of the German Society of Robot-Assisted Urology. The remaining authors declare that the research was conducted in the absence of any commercial or financial relationships that could be construed as a potential conflict of interest.

## Publisher's Note

All claims expressed in this article are solely those of the authors and do not necessarily represent those of their affiliated organizations, or those of the publisher, the editors and the reviewers. Any product that may be evaluated in this article, or claim that may be made by its manufacturer, is not guaranteed or endorsed by the publisher.

## Abbreviations

CAPRA: Cancer of the Prostate Risk Assessment score
CAPRA-S: postsurgical Cancer of the Prostate Risk Assessment score
COVID-19: Coronavirus Disease 2019
HR: high risk
IR: intermediate risk
ISUP: International Society of Urological Pathology
IQR: interquartile range
LR: low risk
LRM: logistic regression model
MRI: magnetic resonance imaging
PCa: prostate cancer
PSA: prostate-specific antigen
QoL: quality of life
RARP: robot-assisted radical prostatectomy
yrs.: years.
